# OncoVar: an integrated database and analysis platform for oncogenic driver variants in cancers

**DOI:** 10.1093/nar/gkaa1033

**Published:** 2020-11-12

**Authors:** Tao Wang, Shasha Ruan, Xiaolu Zhao, Xiaohui Shi, Huajing Teng, Jianing Zhong, Mingcong You, Kun Xia, Zhongsheng Sun, Fengbiao Mao

**Affiliations:** Center for Medical Genetics & Hunan Key Laboratory of Medical Genetics, School of Life Sciences, Central South University, Changsha, Hunan 410083, China; Beijing Institutes of Life Science, Chinese Academy of Sciences, Beijing 100101, China; Department of Clinical Oncology, Renmin Hospital of Wuhan University, Wuhan, Hubei 430072, China; Center for Reproductive Medicine, Department of Obstetrics and Gynecology, Peking University Third Hospital, Beijing 100191, China; Beijing Institutes of Life Science, Chinese Academy of Sciences, Beijing 100101, China; Beijing Institutes of Life Science, Chinese Academy of Sciences, Beijing 100101, China; Key Laboratory of Prevention and Treatment of Cardiovascular and Cerebrovascular Diseases of Ministry of Education, Gannan Medical University, Ganzhou 341000, China; Baiyining Medicine, Beijing 102200, China; Center for Medical Genetics & Hunan Key Laboratory of Medical Genetics, School of Life Sciences, Central South University, Changsha, Hunan 410083, China; CAS Center for Excellence in Brain Science and Intelligences Technology (CEBSIT), Shanghai 200031, China; School of Basic Medical Science, Central South University, Changsha, Hunan 410078, China; Beijing Institutes of Life Science, Chinese Academy of Sciences, Beijing 100101, China; CAS Center for Excellence in Biotic Interactions, University of Chinese Academy of Sciences, Beijing 100049, China; State Key Laboratory of Integrated Management of Pest Insects and Rodents, Chinese Academy of Sciences, Beijing 100101, China; Center of Basic Medical Research, Institute of Medical Innovation and Research, Peking University Third Hospital, Beijing 100191, China

## Abstract

The prevalence of neutral mutations in cancer cell population impedes the distinguishing of cancer-causing driver mutations from passenger mutations. To systematically prioritize the oncogenic ability of somatic mutations and cancer genes, we constructed a useful platform, OncoVar (https://oncovar.org/), which employed published bioinformatics algorithms and incorporated known driver events to identify driver mutations and driver genes. We identified 20 162 cancer driver mutations, 814 driver genes and 2360 pathogenic pathways with high-confidence by reanalyzing 10 769 exomes from 33 cancer types in The Cancer Genome Atlas (TCGA) and 1942 genomes from 18 cancer types in International Cancer Genome Consortium (ICGC). OncoVar provides four points of view, ‘Mutation’, ‘Gene’, ‘Pathway’ and ‘Cancer’, to help researchers to visualize the relationships between cancers and driver variants. Importantly, identification of actionable driver alterations provides promising druggable targets and repurposing opportunities of combinational therapies. OncoVar provides a user-friendly interface for browsing, searching and downloading somatic driver mutations, driver genes and pathogenic pathways in various cancer types. This platform will facilitate the identification of cancer drivers across individual cancer cohorts and helps to rank mutations or genes for better decision-making among clinical oncologists, cancer researchers and the broad scientific community interested in cancer precision medicine.

## INTRODUCTION

The exome constitutes <2% of the human genome but contains ∼85% of known disease-causing variants (1). Early somatic mutations in coding regions can cause developmental disorders (2), whereas progressive accumulation of somatic mutations throughout life can lead to cancer (3). Understanding genetic events that lead to cancer initiation and progression remains one of the biggest challenges in cancer biology. Large cancer sequencing projects, such as The Cancer Genome Atlas (TCGA) and International Cancer Genome Consortium (ICGC), provide unprecedented opportunities to identify causative variants underlying human cancers (4). However, the majority of the somatic missense mutations do not have a noticeable effect (5), and the prevalence of neutral mutations in a cancer cell population impede the distinguishing of cancer-causing driver mutations (6). Dozens of computational algorithms have been developed to predict whether a missense mutation is deleterious or pathogenic based on concepts including evolutionary conservation, structural constraints and the physicochemical attributes of amino acids (7). But cancer driver mutations predicted by these computational methods are lack of consistencies and are prone to false positives (8). Even though we recently have developed a machine learning method to specifically predict cancer-driving deleterious mutations with high accuracy (9), there is still no convenient database to access driver mutations for cancer therapeutic targets.

On the other hand, many approaches have been developed to prioritize cancer driver genes with the advance of next-generation sequencing technologies (10). Most of these tools can be classified into three categories based on three basic principles: (i) frequency-based methods, which consist of identifying genes that are more frequently mutated than the background mutation rate (BMR) (11–16); (ii) subnetwork methods, which identify groups of driver genes based on prior knowledge of networks, such as protein–protein interactions (17–23); (iii) hotspot-based methods (24–26), which are driven by positive selection and are particularly located in functional domains or important residues for 3D protein structures (27,28). However, driver genes predicted from these computational tools also lack consistency since many of these tools are not optimally balanced between precision and sensitivity (10,29). Some of them are overly conservative and missing many true drivers while the others are over-relaxed and yield too many false-positive calls (10). Therefore, discovering a complete catalog of driver genes truly associated with cancer is far from being achieved.

Recently, many databases have been developed to deposit cancer driver genes with the rapid development of prediction methods (30) and the expansion of experimental validations (30,31). For example, the Cancer Gene Census (CGC) is an ongoing project within COSMIC database to catalogue all genes that are causally implicated in cancer through somatic and germline mutations (32). OncoKB is a comprehensive and curated oncology knowledge database with oncologists detailed and evidence-based information about individual somatic mutations and structural alterations present in patient tumors (33). MutPanning provides a resource of driver genes across 28 tumor types with additional driver genes identified according to mutations in unusual nucleotide context (34). Sleeping Beauty Cancer Driver Database (SBCDDB) provides information of cancer driver genes identified in tumor models generated by Sleeping Beauty insertional mutagenesis (35). More and more databases, such as DriverDBv3 (36) and IntOGen (37), are incorporating published prediction approaches to identify driver genes from large-scale cancer projects. Using combinational strategies, consensus-based methods like CTAT (30), ConsensusDriver (38), IntOGen (37) and C3 (39) promise to harness the strengths of different driver prediction methods and provide the best trade-off between sensitivity and specificity. However, there is no available integrated database for convenient search of annotations of driver genes from TCGA and ICGC cancer genomics projects by incorporating cancer driver predictions and prior oncology knowledge.

**Figure 1.**
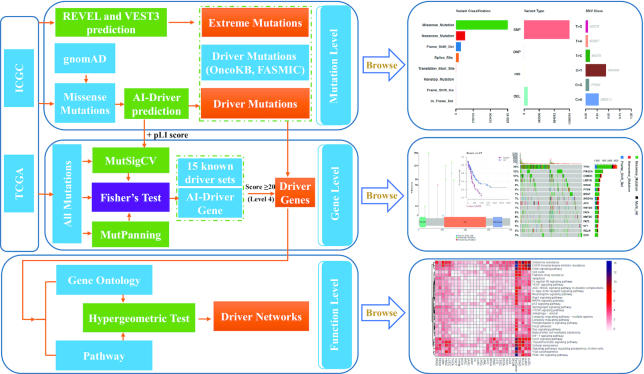
Workflow of OncoVar pipeline. The somatic missense mutations identified from TCGA and ICGC cohorts were used for driver mutation prediction with our recently developed AI-Driver method. Somatic missense mutations with AI-Driver score ≥0.95 and occurred in at least two patients were defined as driver mutations. The MutsigCV, with two additional gene-level covariates – pLI score and maximum AI-Driver score, and MutPanning methods were used for ranking the genes based on the generated P values with all somatic mutations identified from TCGA and ICGC cohorts, respectively. Then, two *P* values were combined by using Fisher method for each gene and further corrected by Benjamini–Hochberg method. Genes with a corrected *P* value ≤0.05 were considered as AI-DriverGenes. AI-DriverGenes combined with other 15 known driver sets were used for gene classification and the pathogenic genes with score ≥20 (Level 4) were considered as driver genes. Only the driver genes with driver or extreme mutations were used for gene-level exploration. Finally, driver genes were used to identify driver pathways (FDR < 0.05) by using the hypergeometric test.

To satisfy these demands, we developed a database, called ONCOVAR (Figure 1), to discover ONCOgenic driver VARiants from large cancer sequencing projects by employing published bioinformatics algorithms and incorporating known driver events. OncoVar provides four points of view, ‘Mutation’, ‘Gene’, ‘Pathway’ and ‘Cancer’, to help researchers to visualize the relationships between cancers and driver variants. Our database provides a valuable resource for cancer studies by presenting cancer-causing mutations, mutated driver genes and oncogenic signaling pathways. The findings based on our database highlight the importance of combination of algorithm predictions and prior knowledge for the interpretation of pathogenic variants in human cancers and complex diseases.

## MATERIALS AND METHODS

### Somatic mutation collection

OncoVar includes unbiased interpretation of 2 605 700 somatic mutations from the entire 10 769 tumor sample datasets of 33 cancer types by harmonizing the results of seven algorithms, yielded by the uniform analysis of all TCGA exome data by the Multi-Center Mutation-Calling in Multiple Cancers (MC3) network (40) (https://api.gdc.cancer.gov/data/1c8cfe5f-e52d-41ba-94da-f15ea1337efc). To reduce the false-positive rate for driver gene discovery, we implemented three strategies to optimize driver detection and data quality. Briefly, we excluded 344 hypermutated tumors because of artifact sensitivity to high background mutation rates. All mutations that passed the MC3 filter criteria were included. Finally, samples marked with inconsistent pathology were excluded. Clinical information on TCGA was downloaded from the Genomic Data Commons Data Portal (https://portal.gdc.cancer.gov/). Moreover, we curated 23 159 591 somatic mutations from 1942 samples of 18 cancer types from the ICGC Data Portal (https://dcc.icgc.org/api/v1/download?fn=/PCAWG/consensus_snv_indel/final_consensus_passonly.snv_mnv_indel.icgc.public.maf.gz).

### Germline mutations from normal population

Over 270 million variants in the gnomAD v2.1 dataset (https://gnomad.broadinstitute.org) have been widely used as a resource for allele frequency estimates in the context of rare disease, which can improve power for disease gene discovery and exploring the biological impact of genetic variation (41,42). We aggregated 14 967 411 mutations from 125 748 control-only reference individuals after removing mutations without ‘PASS’ in the filtering criterion. Gene-level pLI scores of all genes were downloaded from gnomAD database. Meanwhile, we collected gene-level values of loss-of-function observed/expected upper bound fraction (LOEUF) calculated by using 141 456 human genomes from gnomAD database.

### Identification of extreme and driver mutations

Similar to our previous studies (2,4,43–48), we performed ANNOVAR (49) to annotate all somatic mutations with respect to variant-level data sources, including the following information: (i) functional effects of variants; (ii) functional prediction of missense mutations by 23 predictive algorithms; (iii) allele frequencies in different populations; (iv) reported variants in different disease- and phenotype-related databases and (v) some other genome features, such as CytoBand. LoF variants, including stop-gain, stop-loss, splicing site SNVs, frameshift indels and deleterious missense somatic mutations were regarded as potential extreme variants. Similar to our previous study (31), we obtained predictive scores and the pathogenicity consequences of missense variants from 23 in silico algorithms or tools, including SIFT (50), PolyPhen2-HDIV (51), PolyPhen2-HVAR (51), LRT (52), MutationTaster (53), MutationAssessor (54), FATHMM (55), PROVEAN (56), MetaSVM (57), MetaLR (57), VEST3 (58), M-CAP (59), CADD (60), GERP++ (61), DANN (62), fathmm-MKL (63), Eigen (64), GenoCanyon (65), fitCons (66), PhyloP (pP100way) (67), PhastCons (pC100way) (68), SiPhy (69) and REVEL (70) (Supplementary Table S1). To facilitate interpretation, we presented the final predictive scores of ReVe as percentiles that reflect the relative rank of pathogenicity for missense variants according to our recently published study (71), with the lowest score (i.e., 0.00) being the most benign variant and the highest score (i.e. 1.00) being the most deleterious variant. Extreme mutations were defined by loss of function mutations and missense mutations with a ReVe score ≥0.5. Then, we employed our recently developed machine-learning method, AI-Driver , to determine candidate driver mutations by testing missense mutations with the model trained by using the features of known driver mutations. The missense mutations with AI-Driver score ≥0.95 and occurred in at least two patients were defined as driver mutations in each cancer cohort.

### Identification of AI-DriverGene

AI-DriverGene is prioritized by combining predictions of MutSigCV (72) and MutPanning (34) methods. First, MutSigCV was employed to generate the *P* value of each gene with two additional covariates including gene-level pLI score and maximum AI-Driver score of mutations within each gene. Then, the MutSigCV *P*-value was combined with the MutPanning *P*-value using the Fisher method. Finally, the joint *P* values were corrected by Benjamini-Hochberg and genes with a corrected *P* threshold of 0.05 were considered as AI-DriverGenes.

### Five-tiered consensus-based classification of driver genes

We used a consensus-score for the classification of driver genes. Consensus-score is based on an improved Borda approach, where each gene was given a score equal to the sum across all driver sets of its weight (38). Totally, 16 driver sets (SupplementaryTable S2) were used for consensus-score calculation and these driver sets were further classified into two groups including Group1 involving four gold standard driver sets (CGC, OncoKB, AI-DriverGene and MutPanning) and Group2 containing the remaining twelve driver sets (https://oncovar.org/welcome/links).

(1) $$\begin{equation*}Score\ \left( {gen{e_i}} \right) = \sum\nolimits_{j = 0,\ \ \ over\ 16\ driver\ sets}^{j = 16} {{\rm{weigh}}{{\rm{t}}_j}\left\{ {\begin{array}{@{}*{1}{c}@{}} {10,\ j \in Group1\ \ }\\ {1,\ j \in Group2} \end{array}} \right.} \end{equation*}$$

Genes covered by neither of these 16 driver sets were assigned 0 and then all other genes were ranked according to this score. Furthermore, to make the gene ranking to a standardized and easily interpretable format, we introduced the following procedures to classify all genes into four grades from non-pathogenic to pathogenic): Level 0 (non-pathogenic, score = 0), Level 1 (possible pathogenic, score = 1), Level 2 (likely pathogenic, 1 < score ≤ 10), Level 3 (probable pathogenic, 10< score < 20) and Level 4 (pathogenic, score ≥ 20). Only the pathogenic genes in Level 4 were considered as driver genes and used for downstream analysis.

### OncoVar scoring system based on Gaussian model

To systematically compare the ‘driverness’ of each mutation, gene and pathway, we calculated the OncoVar score using Gaussian model (2) based on *x* = –log_2_(FDR), in which σ represents the standard deviation of *x* while μ represents the expected value of *x*. An OncoVar score of *x* is the cumulative probability of X ≤ *x* following *Z*-score transformation. A higher OncoVar score (0–1) represents a greater ability of cancer initiation and progression by the mutation, gene or pathway.

(2) $$\begin{equation*}f\left( {\chi ;\mu ,\sigma } \right) = \ \frac{1}{{\sigma \sqrt {2\pi } }}\,\int_{{ - \infty }}^{\chi }{{\exp \ \left( { - \frac{{{{\left( {\chi - \mu } \right)}^2}}}{{2{\sigma ^2}}}} \right)d\chi }}\end{equation*}$$

### Essential and nonessential genes in human cancers

We curated 7168 essential and 21 373 nonessential genes from the OGEE v2 database (73). Then, we calculated the mean expression of these genes in 33 tumor types from TCGA cohorts. We filtered the essential genes with a mean TPM (Transcripts Per Kilobase Million) value < 10, while we filtered the nonessential genes with a mean TPM value >1 in 33 human cancer types. Finally, we obtained 6197 and 903 high-confidence essential and nonessential genes in human cancers, respectively (Supplementary Table S3).

### Gene Ontology enrichment analyses

Gene enrichment was performed using the R package clusterProfiler version 3.2.14 (74). Each cluster from the driver genes was compared with the background of all other genes sequenced at sufficient depth in our study, with a Benjamini–Hochberg FDR threshold of 0.05 as significant enrichment. Enrichment of KEGG pathways was analyzed with the enrichKEGG function.

### Profile and visualization of cancer driver events

We employed Maftools to summarize, analyze and annotate MAF files in an efficient manner from either TCGA or ICGC sources (75). In detail, we used ‘plotmafSummary’ to plot the summary of the MAF file, which displays the number of variants in each sample as a stacked barplot and variant types as a boxplot summarized by Variant_Classification. Better representation of the MAF file was shown as oncoplots. Side barplot and top barplots were controlled by drawRowBar and drawColBar arguments, respectively. The titv function classifies SNPs into transitions and transversions and returns a list of summarized tables. Summarized data were visualized as a boxplot showing overall distribution of six different conversions and as a stacked barplot showing the fraction of conversions in each sample. Lollipop plots are a simple and effective way to show mutation spots on protein structure. Many oncogenes have preferential sites which are mutated more often than any other locus. These spots are considered to be mutational hot-spots and lollipop plots are used to display them along with the rest of the mutations. We drew such plots using the function lollipopPlot in Maftools. In addition, we plotted word cloud plots for mutated genes with the function geneCloud. The size of each gene is proportional to the total number of samples in which it is mutated.

### Mutually exclusive and co-occurring driver genes

Many disease-causing genes in cancer are co-occurring or show strong exclusiveness in their mutation pattern. Such mutually exclusive or co-occurring sets of genes can be detected using the somaticInteractions function in Maftools, which performs a pairwise Fisher's exact test to detect such a significant pair of genes. The somaticInteractions function also uses cometExactTest to identify potentially altered gene sets involving > two genes. The top 50 driver genes were used to perform exclusive/co-occurrence analysis and labeled with a *P* value threshold of 0.05 and 0.01.

### Survival analysis

The function mafSurvive in Maftools was used to perform survival analysis and draw a Kaplan–Meier curve by grouping samples based on the mutation status of driver genes or manually provided samples that make up a group.

### Drug–gene interactions

Numerous drugs that are already approved for specific diseases have known protein targets, which may be relevant for other disease types as well. A systematic identifying druggable genes in various diseases would help streamline the process of developing new drugs for these targets, even if no specific drugs are available for them yet (76). Therefore, we integrated the data for drug–gene interactions and gene druggability from the Drug–gene Interaction Database (DGIdb 3.0) (77) to assist with precision medicine in cancer treatment.

## RESULTS

### Website interface and search results

OncoVar provides four points of browse, ‘Mutation’, ‘Gene’, ‘Pathway’ and ‘Cancer’, to help researchers to visualize the relationships between cancers and driver variants for each cancer type and pan-cancer cohort. Firstly, users could search driver/extreme mutations by inputting variants, dbSNP ids or genomic regions based on human GRCh37/hg19 genome. Outputs of mutation search include OncoVar scores, six driver mutation annotations and 23 pathogenicity scores. Secondly, users could search driver genes by inputting gene symbols, Ensembl gene ids or Entrez ids. Outputs of gene search include consensus score, driver level, OncoVar scores, targeting drugs and 18 additional driver gene annotations coupled with lollipop plot of associated mutations and survival plot of clinical data. Thirdly, users could search oncogenic pathways by inputting GO and KEGG ids or GO and KEGG names. Outputs of pathway search include OncoVar scores and associated genes. Finally, OncoVar provides comprehensive summaries of driver/extreme mutations, driver genes and oncogenic pathways for each cancer type and pan-cancer cohort. The summaries of driver/extreme mutations include plot of mutation classification and plot of transition and transversions. The summaries of driver genes include waterfall plot of top 30 mutated genes, interaction plot and GeneCloud plot. The summaries of oncogenic pathways include KEGG dotplot, biological process dotplot, cellular component dotplot, molecular function dotplot and biological process GOgraph (Figure 1, Supplementary Figure S1). In order to improve the robustness of our database, we developed four convenient functions including ‘Batch mutation annotation’, ‘Cancer specific pathway enrichment’, ‘Hg19ToHg38 conversion’ and ‘Hg38ToHg19 conversion’ in drop down box of ‘Analysis’ column. Especially, the function of ‘Cancer specific pathway enrichment’ enables to perform GO and KEGG enrichment analysis based on the uploaded input genes by users. This enrichment analysis implements a hypergeometric test restricted to the identified driver genes in each GO/KEGG item for each cancer type as well as pan-cancer cohort. To our knowledge, our platform is the first webserver which could perform enrichment analysis of cancer-specific driver pathway in individual cancer types as well as pan-cancer cohort.

### The features of cancer driver mutations

**Figure 2.**
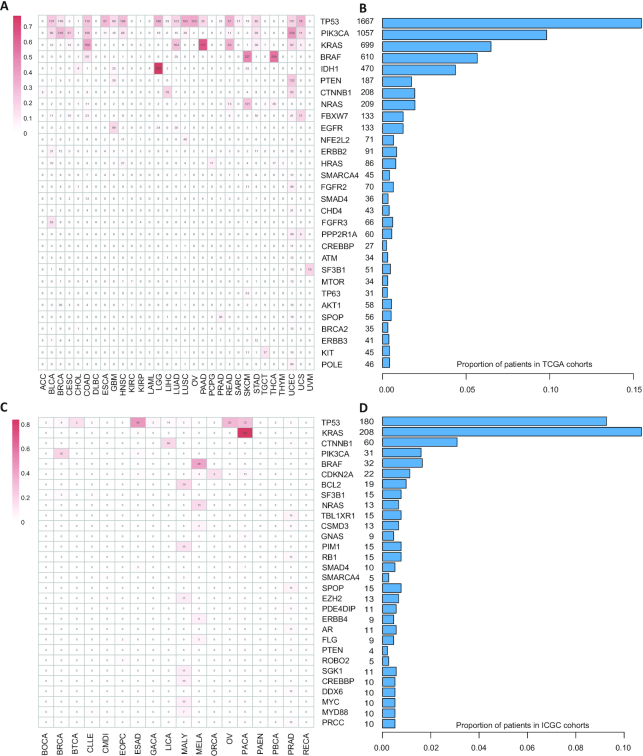
A snapshot of driver mutations identified from TCGA and ICGC cohorts. (A, C). Top 30 enriched driver genes by the driver mutations across each cancer type from TCGA cohorts (**A**) and ICGC cohorts (**C**). (B, D) TP53 is the top one mutated gene across all cancers and detected with driver mutations in ∼23% and ∼17% patients from TCGA (**B**) and ICGC (**D**) cohorts, respectively.

By employing our method AI-Driver, we identified 16 923 and 3409 driver missense mutations from TCGA and ICGC cohorts, respectively. We then classified the driver mutations into five groups according to consensus score based on four known driver sets and AI-Driver prediction. We found the proportions of driver mutations of CGC increased in groups with higher consensus score (Supplementary Figure S2A, B). To symmetrically evaluate the performance of predictions by AI-Driver, we employed receiver operating characteristic (ROC) analysis benchmarked by four known driver sets. We found driver predictions by AI-Driver had a superior and stable performance with high area under the ROC curve (AUC) value (Supplementary Figure S2C, D). The AUC values for TCGA cohorts were 0.972, 0.959, 0.969 and 0.966 while the AUC values for ICGC cohorts were 0.968, 0.958, 0.967 and 0.969, benchmarked by CGI (78), FASMIC (31), OncoKB (33) and PMID25348012 (79), respectively. All of these newly detected driver mutations by AI-Driver were integrated into our OncoVar database. More than 46% patients in TCGA cohorts had at least one driver mutations in driver gene TP53, PIK3CA, KRAS, BRAF, IDH1, PTEN, CTNNB1 and NRAS (Figure 2A, B) while more than 29% patients in ICGC cohorts suffered from at least one driver mutations in driver gene TP53, KRAS, CTNNB1 and PIK3CA (Figure 2C, D). Top 30 driver genes and all genes harbored driver mutations were showed in Figure 2 and supplemental table 4, respectively. Therefore, OncoVar provides access to search all newly detected and known driver mutations in each cancer type and pan-cancer cohort from TCGA and ICGC projects.

### The landscape of cancer driver genes

**Figure 3.**
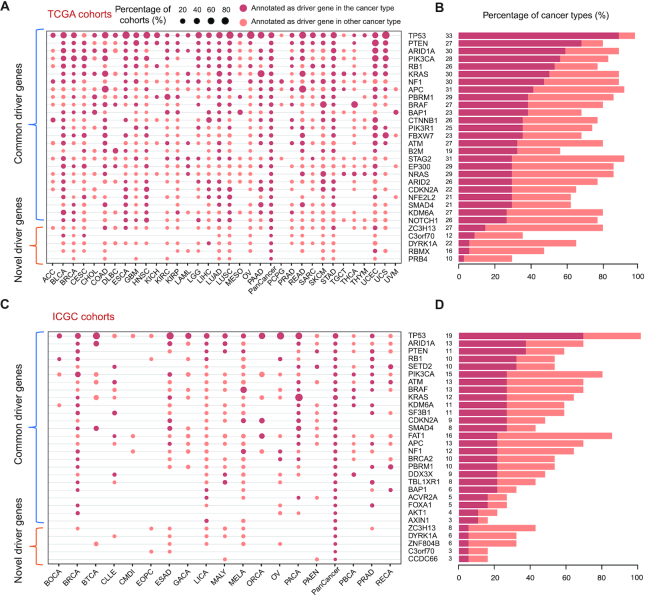
A snapshot of driver genes identified in TCGA and ICGC cohorts. (**A, C**). The range of cancer types with 25 exemplary common driver genes and five exemplary novel driver genes represented as dots. The size of the dots represents the percentage of all cohorts of the cancer type in which the gene is identified as a driver and with driver or extreme mutation. (**B, D**). The number and percentage of cancer types in which each gene appears as a driver in all cancer types is represented in the bars.

**Figure 4.**
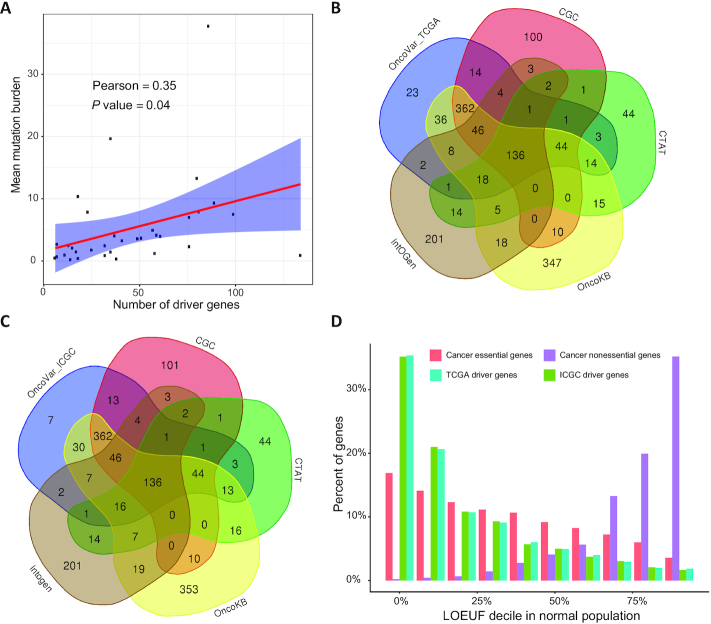
Features of identified driver genes. (**A**). A significant positive correlation between average mutation burden in a cancer type and the number of identified driver genes. (**B, C**). OncoVar identified many known driver genes which are consistent with results by other methods including CGC (49), CTAT (31), OncoKB (50), IntOGen (37) and several novel driver genes. (**D**). Percent of genes among different loss-of-function genes observed/expected upper bound fraction (LOEUF) (28) in a normal population from gnomAD.

By employing the OncoVar pipeline, we identified 713 and 686 driver genes from TCGA and ICGC pan-cancer cohorts, respectively. Specially, we identified 806 and 696 driver genes from TCGA and ICGC individual cancer types, respectively. The number of cancer driver genes varies among cancer types, with Uveal Melanoma (UVM having the fewest (6 genes) and Acute Myeloid Leukemia (LAML) having the most (134 genes) from TCGA cohorts (Supplementary Table S5). Our results revealed that the roles of driver genes across cancer types is much more widespread than previously documented (Figure 3). For example, the pattern of somatic mutations in ataxia telangiectasia mutated (ATM) shows signals of positive selection across 11 and 5 tumor types in TCGA and ICGC cohorts, respectively (Figure 3). However, it is annotated in the CGC only as a driver of T-cell-prolymphocytic leukemia. In addition, we observed a moderate positive correlation (Pearson's R = 0.35, *P* value = 0.04) between mean mutation burden in a cancer type and the number of identified driver genes (Figure 4A; Supplementary Table S6). Moreover, OncoVar identified 23 and 7 novel driver genes from TCGA and ICGC pan-cancer cohorts, respectively, compared with four well-known databases including CGC (80), OncoKB (33), IntOGen (37) and CTAT (30) (Figure 4B, C). Furthermore, we collected the LOEUF value for each gene calculated by using 141,456 human genomes from gnomAD (41). High LOEUF scores suggest a relatively higher tolerance to inactivation while low LOEUF scores indicate strong selection against predicted loss-of-function variation in a given gene. The distribution of LOEUF of driver genes from both TCGA (mean LOEUF = 0.5062) and ICGC (mean LOEUF = 0.5028) pan-cancer cohorts maintain the same trend with that of cancer essential genes (mean LOEUF = 0.7533) but not cancer nonessential genes (mean LOEUF = 1.5739) (Figure 4D). The mean LOEUF values of TCGA driver genes, ICGC driver genes and cancer essential genes are significantly lower than that of cancer nonessential genes (Mann–Whitney *U* test, *P* < 2.2e–16). Our results indicated that cancer driver genes are much more constraint than cancer nonessential genes in the human population and these driver genes are essential for carcinogenesis in human cancers. To demonstrate the functions of the novel driver genes identified by OncoVar, we employed the DepMap database (https://depmap.org/portal/) to further investigate whether these novel driver genes are cancer dependency genes. Interestingly, we found 19 out of 23 (82.6%) and 6 out of 7 (85.7%) novel driver genes from TCGA and ICGC pan-cancer cohorts are related to cancer vulnerabilities of at least one kind of cancers which were validated by CRISPR or RNAi knockout libraries, respectively (Supplementary Table S7).

### The panorama of oncogenic pathways

**Figure 5.**
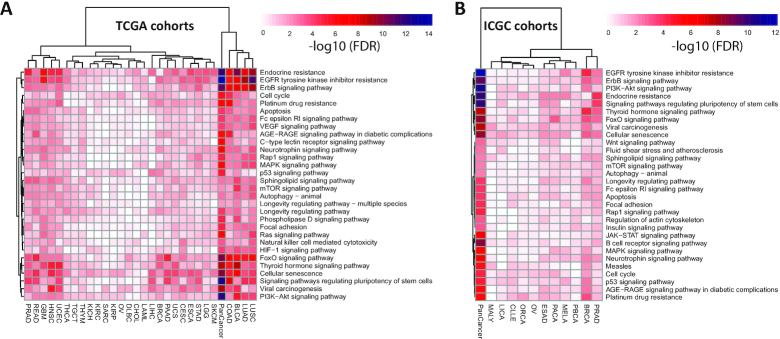
Top 30 enriched KEGG pathways in TCGA (**A**) and ICGC (**B**).

Genetic alterations in signaling pathways that control cell-cycle progression, apoptosis, and cell growth are common hallmarks of cancers (81). However, other kinds of signaling pathways and gene ontologies (GOs) are rarely investigated. Briefly, we identified 1941 and 1919 GOs and KEGG pathways from TCGA and ICGC pan-cancer cohorts (Supplementary Table S8). Consistent with a previous study (82), we found that enriched GOs and pathways are shared across anatomical origins and cell types in both TCGA and ICGC cohorts (Supplementary Figure S3). For instance, the top 30 enriched pathways ranked by FDR in TCGA pan-cancer include well-known cancer pathways such as the ErbB signaling pathway, cell-cycle regulation, apoptosis regulation, p53 signaling pathway, MAPK signaling pathway, mTOR signaling pathway, Ras signaling pathway, PI3K-Akt signaling pathway, regulation of mitotic cell cycle and epithelial cell proliferation (Figure 5A, Supplementary Figure S4A). Similarly, top 30 functional terms in ICGC pan-cancer contain common cancer pathways such as PI3K–Akt signaling pathway, Wnt signaling pathway, mTOR signaling pathway, B cell receptor signaling pathway, JAK-STAT signaling pathway, G1/S transition of mitotic cell cycle, lymphocyte differentiation and histone modification (Figure 5B, Supplementary Figure S4B). Some pathways were mutated across most of the cancer types while other pathways were more specific to specific tumor types. Our platform OncoVar provides enrichment summary and search of GO and pathways in each cancer type and pan-cancer cohort from TCGA and ICGC projects.

### Mutually exclusive and co-occurring driver genes

Mutually exclusive patterns between alterations across large patient cohorts have been associated with functional redundancy, indicating that once one occurred, the second will not provide a further selective advantage, or alternatively indicating that cells cannot survive with both alterations with synthetic lethality. On the other hand, co-occurrence patterns of alterations in many tumor samples indicate functional synergies and may reflect therapeutic resistance targeting one of the alterations (81). We employed Maftools to explore significantly mutually exclusive and co-occurring driver genes for pan-cancer and each cancer cohort. Among the top 50 cancer driver genes in TCGA pan-cancer cohorts, we found that the TP53, IDH1 and BRAF gene is most likely to be mutually exclusive with other altered driver genes (Supplementary Figure S5 and S6), indicating that oncogenic alteration in one of these three genes is sufficient to carcinogenesis. In terms of co-occurrence patterns, we found that the rest of driver genes, such as NOTCH1, EGFR, PIK3CA, MTOR and EP300 gene are most likely to be co-mutated with other driver genes. Our results indicated that drug combinations may improve efficient treatments based on the occurrence of actionable alterations across different tumor types. Our platform OncoVar provides mutually exclusive and co-occurring patterns of top 50 driver genes in each cancer type and pan-cancer cohort from TCGA and ICGC projects.

### The landscape of targeted therapies

**Figure 6.**
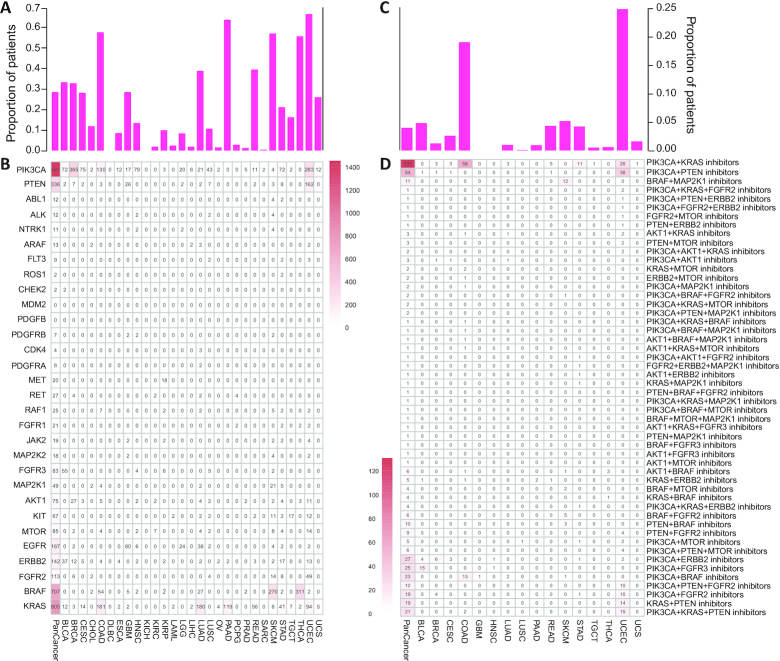
Driver genes and their candidate actionable genes in TCGA. (**A**). Cumulated proportion of patients of all the thirty driver genes identified by OncoVar and annotated as the candidate actionable genes by OncoKB for each cancer type. (**B**). The number of patients for each cancer type in which the gene is identified as a driver and with driver or extreme mutation. (**C**). Cumulated proportion of patients with multiple candidate actionable gene combinations for each cancer type. (**D**). The number of patients for each cancer type with multiple candidate actionable gene combinations.

The development of therapies targeting altered driver proteins provides the promise of precision cancer medicine (83). We identified 30 driver genes predicted to be clinically actionable genes by OncoKB (33) in 29 cancer types from TCGA cohorts (Figure 6A, B). These driver genes harbored different numbers of driver mutations in different cancer types. For instance, PIK3CA, KRAS and BRAF harbored 1457, 805 and 707 driver mutations from pan-cancer cohorts, respectively. Specially, PIK3CA gene had 365 and 283 driver mutations in breast invasive carcinoma (BRCA) and thyroid cancer (THCA), respectively. In addition, BRAF gene had 270 and 311 driver mutations in skin cutaneous melanoma (SKCM) and THCA, respectively. The KRAS gene had 181 and 180 driver mutations in colon adenocarcinoma (COAD) and lung adenocarcinoma (LUAD), respectively. PTEN gene has 162 driver mutations in uterine corpus endometrial carcinoma (UCEC). Moreover, we identified 28 driver genes that harbor potentially actionable mutations annotated by OncoKB database. 28.78% of tumors had multiple targetable driver genes in TCGA pan-cancer cohorts (Figure 6A), indicating opportunities for combination therapy, which is consistent with a previous study (81). We then explored combination therapeutic opportunities based on the actionable mutations which were currently approved for clinical therapies or investigational therapies. Intriguingly, we revealed 148 kinds of potential combination therapies within all actionable driver genes in 19 cancer types (Supplementary Table S9). Specially, we revealed 52 kinds of potential combination therapies within top 10 actionable driver genes, including 22 tri-combinations and 30 bi-combinations in 16 cancer types (Figure 6C, D). We found that the COAD and UCEC cohorts have the most possibilities for combination therapies within 19.17% and 25.00% patients, respectively (Figure 6C). COAD has 13 possible therapy combinations such as inhibition of PIK3CA+KRAS and PIK3CA+BRAF, while UCEC has 18 possible therapy combinations such as PIK3CA + KRAS and PIK3CA + PTEN (Figure 6D). In addition, we validated these 30 actionable driver genes in 11 cancer types from ICGC cohorts (Supplementary Figure S7). Continuous update of drug–gene interaction in OncoVar will improve the *in-silico* prescription based on actionable cancer drivers, thus increasing additional targeting opportunities in personalized cancer medicine.

## DISCUSSION

Recent studies began to pay efforts to systematically investigate potential driver mutations (84), cancer driver genes (30) and oncogenic signaling pathways (81) in different cancer types. Several studies evaluated the performance of existing tools for predicting cancer-causing mutations, but the results showed that identifying oncogenic driving mutations remains a significant challenge (8,79). Herein, we employed our recently developed method AI-Driver, which integrates 23 pathogenicity scores using machine learning algorithm, to determine the ‘driverness’ level of a somatic mutation. Rapid progress has been made in computational approaches to prioritize cancer driver genes. Nevertheless, driver gene lists predicted from these computational tools lack consistency and are prone to false positives (10). Current research is far from achieving the ultimate goal of discovering a complete catalog of driver mutations and genes truly associated with human cancers.

Currently, several databases and frameworks have been developed to integrate driver genes from large-scale genomic data (2), such as DriverDBv3 (36) and IntOGen (85). DriverDBv3 is a multi-omics database for cancer driver gene research which applies published bioinformatics algorithms to determine driver genes along with molecular features and provides an informative visualization of integrative cancer omics data (36). IntOGen is a framework for systematic and automatic identification of mutational driver genes across tumor types (85). Nevertheless, these platforms are not convenient to simultaneously annotate and prioritize tens of thousands of somatic mutations and mutated genes detected by large-scale genomic sequencing. DriverDBv3 predicted driver genes based on the individual criteria of various algorithms but did not combine the driver gene predictions into a consensus ranking (36). The IntOGen framework employed a weighted method to combine driver predictions of seven methods to render a consensus ranking of genes while IntOGen did not integrate known driver mutations and genes from other resources. Whereas, OncoVar employed a new strategy to combine driver predictions as well as known driver genes and predicted driver mutations by our recently developed method AI-Driver. OncoVar is an integrated database to systematically prioritize the oncogenic ability of somatic mutations and mutated genes detected from large cancer sequencing projects. Herein, we have analyzed driver events from TCGA and ICGC sequencing projects in the current release while the collection of driver events from other cancer projects/literatures will be updated regularly in the future.

Apart from mutational driver events by coding point mutations, copy-number alterations (CNAs) and rearrangements can also act as cancer drivers in cancer development though nearly 80% of cancer patients harbored at least one mutational driver events (86). In this database, we purposefully focused on missense driver mutations affecting protein-coding genes instead of CNAs or rearrangements. On the other hand, large number of non-coding somatic mutations are linked to regulatory networks (4) though non-coding *cis*-regulatory driver mutations in known cancer genes are much less frequent than protein-coding ones (87). Moreover, our previous studies demonstrated that synonymous mutations involved in posttranscriptional dysregulation and enhancer regions involved in epigenome remodeling could contribute to the etiology of human cancers (88,89). Therefore, we need to pay more attention to driver events occurred in not only coding regions but also non-coding regions in the future research.

In summary, OncoVar is a useful platform which employs published bioinformatics algorithms to describe mutational driver patterns of large-scale genomic sequencing datasets. First, we identified 16 923 and 3409 highly confident driver mutations from the TCGA and ICGC cohorts by using our machine learning method AI-Driver, respectively. Second, we identified 806 and 696 driver genes from TCGA and ICGC individual cancer types, respectively. Third, we determined 1941 and 1919 GO/KEGG pathways from TCGA and ICGC individual cancer types, respectively. Finally, drug annotations of driver genes provide opportunities of bi-combination and tri-combination therapy in some kinds of cancer types. To our knowledge, OncoVar is the first integrated database which was designed to explore the driver events and interpret their putative mechanism of carcinogenesis across tumor types by incorporating cancer driver predictions and prior oncology knowledge. OncoVar aids the identification of drivers across tumor types and helps rank mutations or genes for better decision-making for the clinical and scientific community interested in cancer precision medicine. We are dedicated to maintaining and improving OncoVar since it is a useful resource for both research and clinical community.

## Supplementary Material

gkaa1033_Supplemental_Files
