# Supplementary figures and images for: OncoVar: an integrated database and analysis platform for oncogenic driver variants in cancers
Source: Nucleic Acids Res. 2020 Nov 12;49(D1):D1289–301. doi: 10.1093/nar/gkaa1033 (PMC7778899; doi:10.1093/nar/gkaa1033)

Supplemental Figure 1

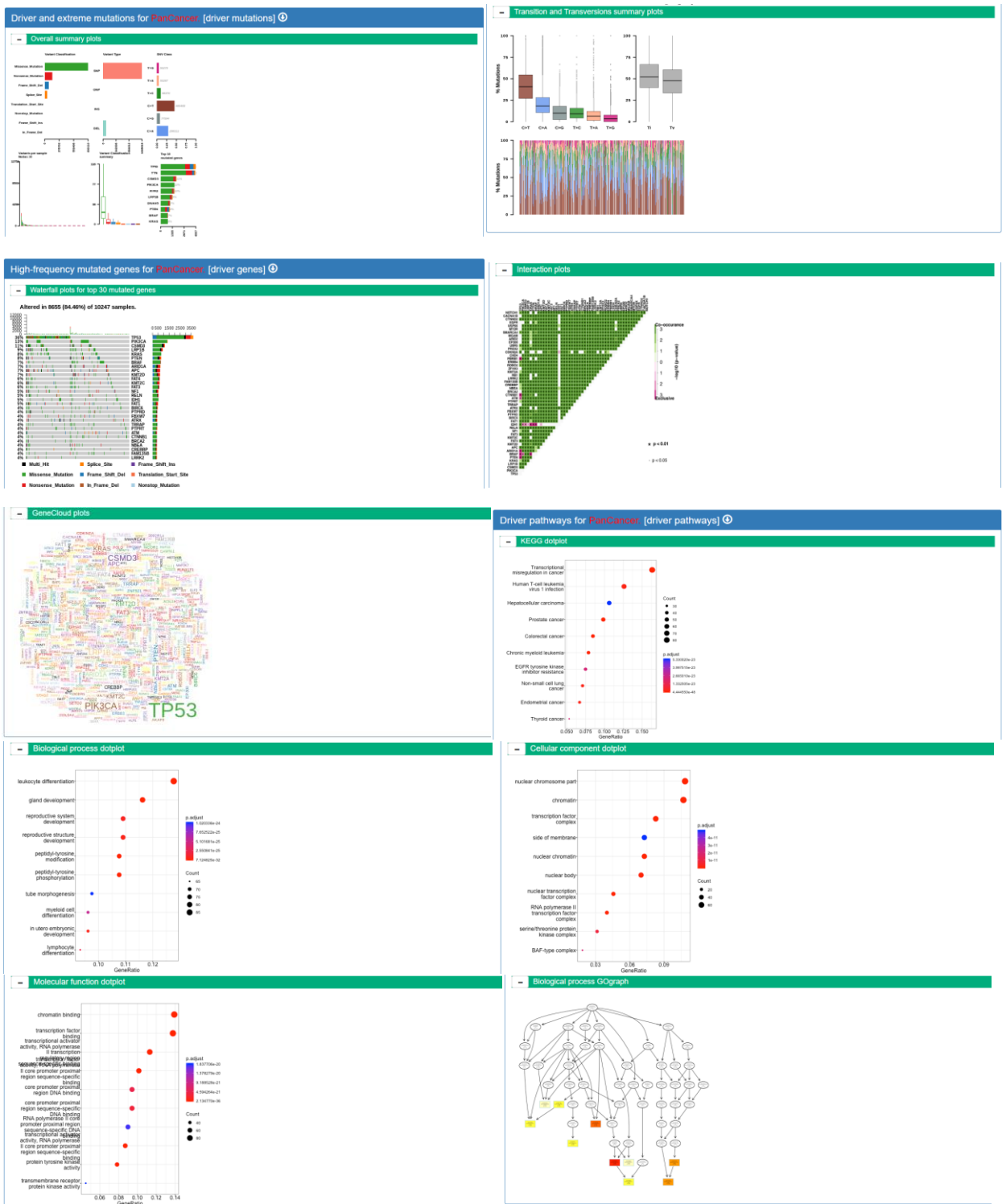

Supplement: gkaa1033_Supplemental_Files [file gkaa1033_supplemental_files.zip › Figure S1 web interface.pdf]

# Supplemental Figure 2

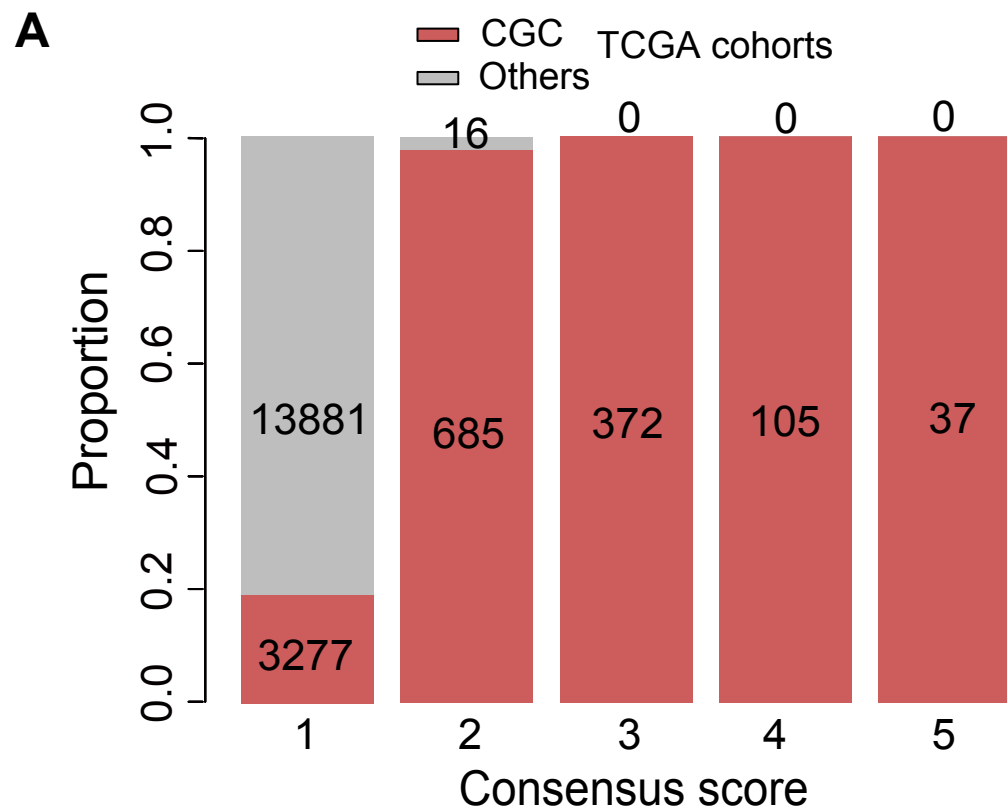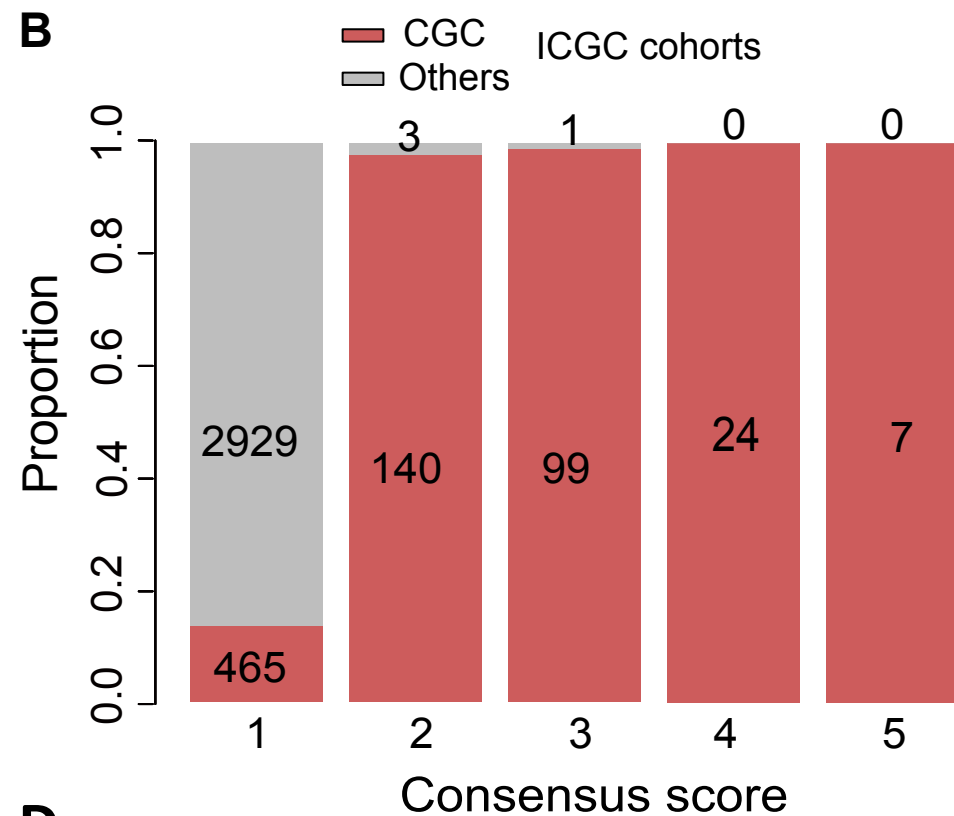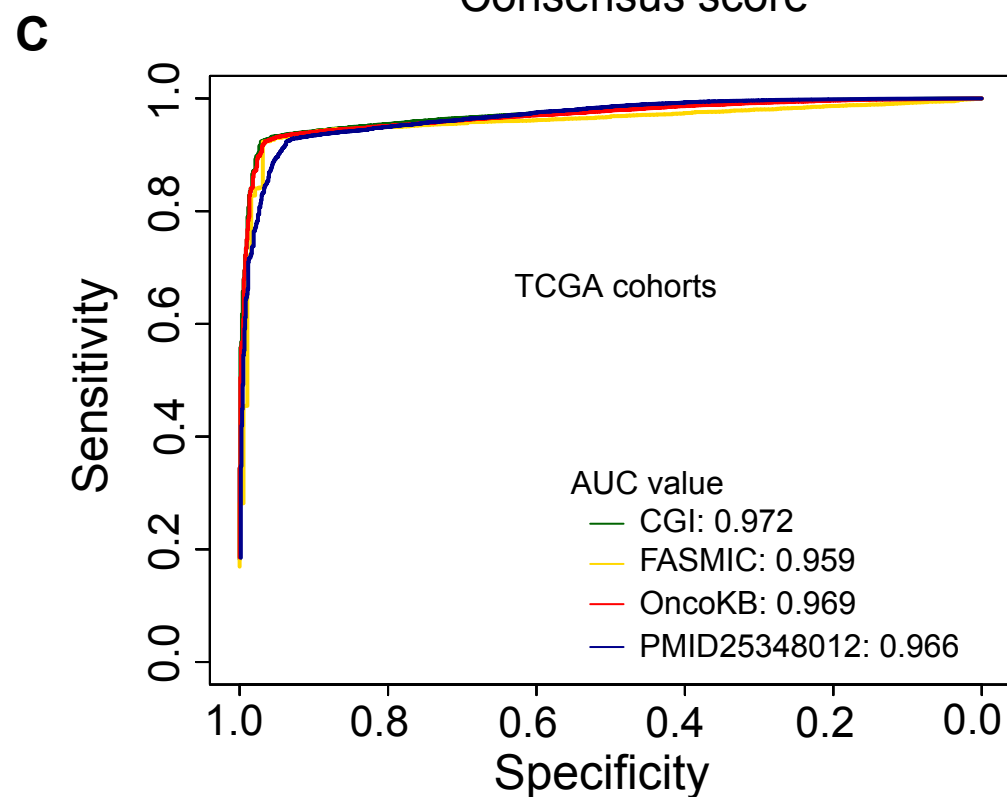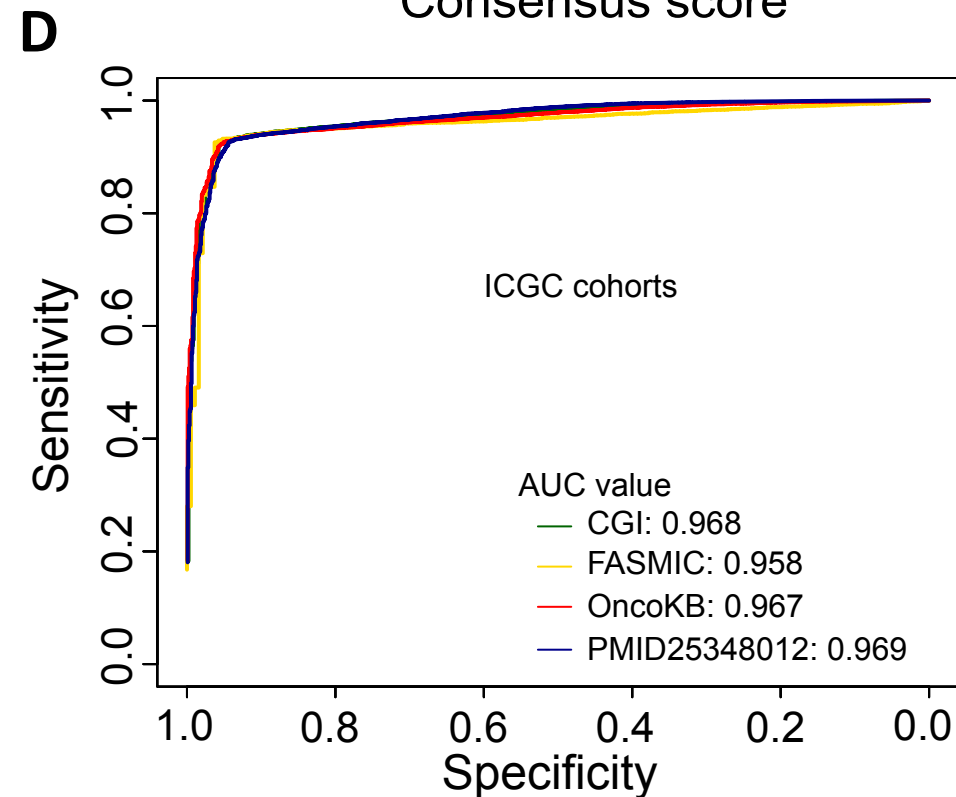

Supplement: gkaa1033_Supplemental_Files [file gkaa1033_supplemental_files.zip › Figure S2. driver.mutation.barplot.ROC-xiaolu+updated_Tania_modified.pdf]

Supplemental Figure 3

A

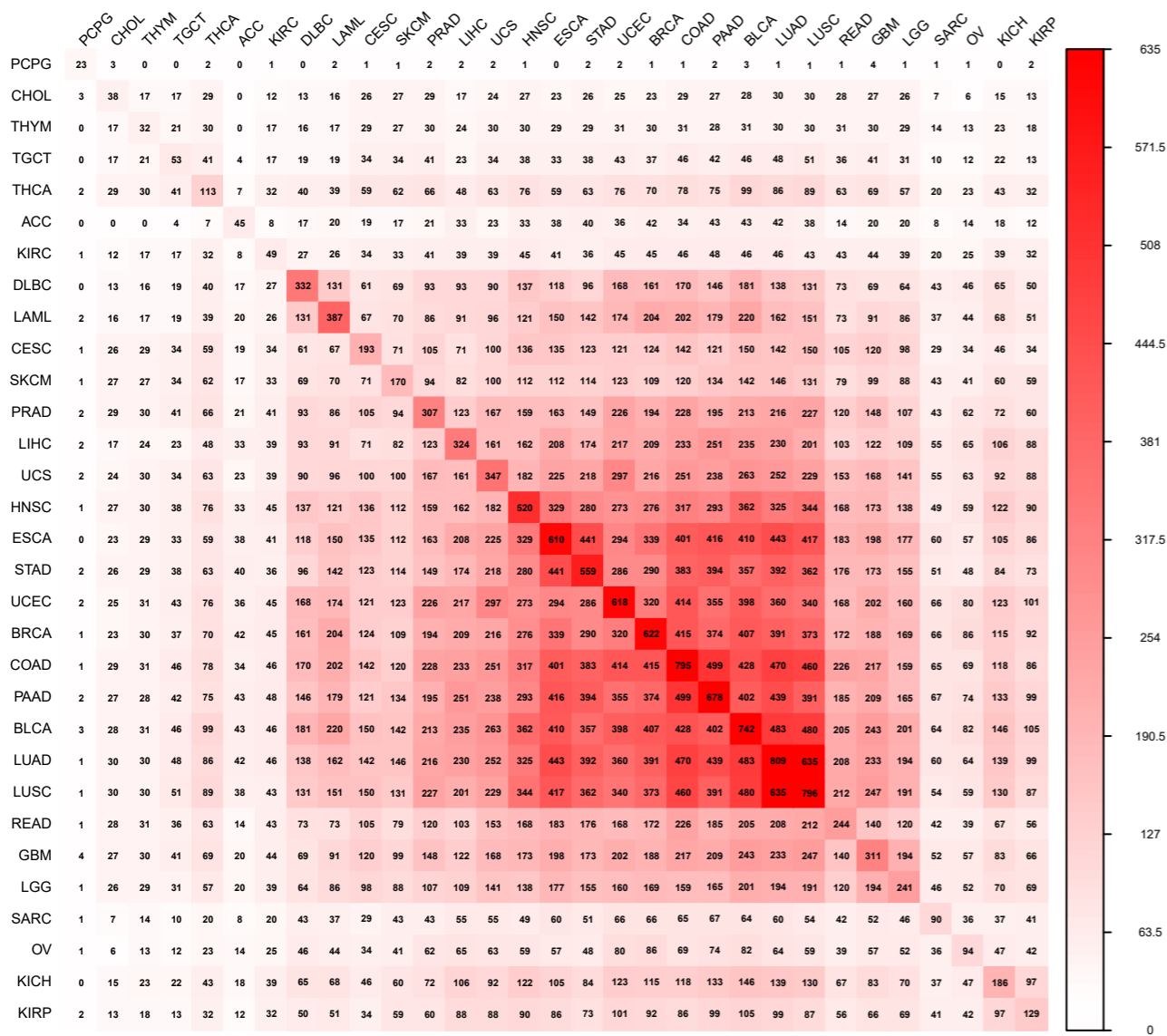

B

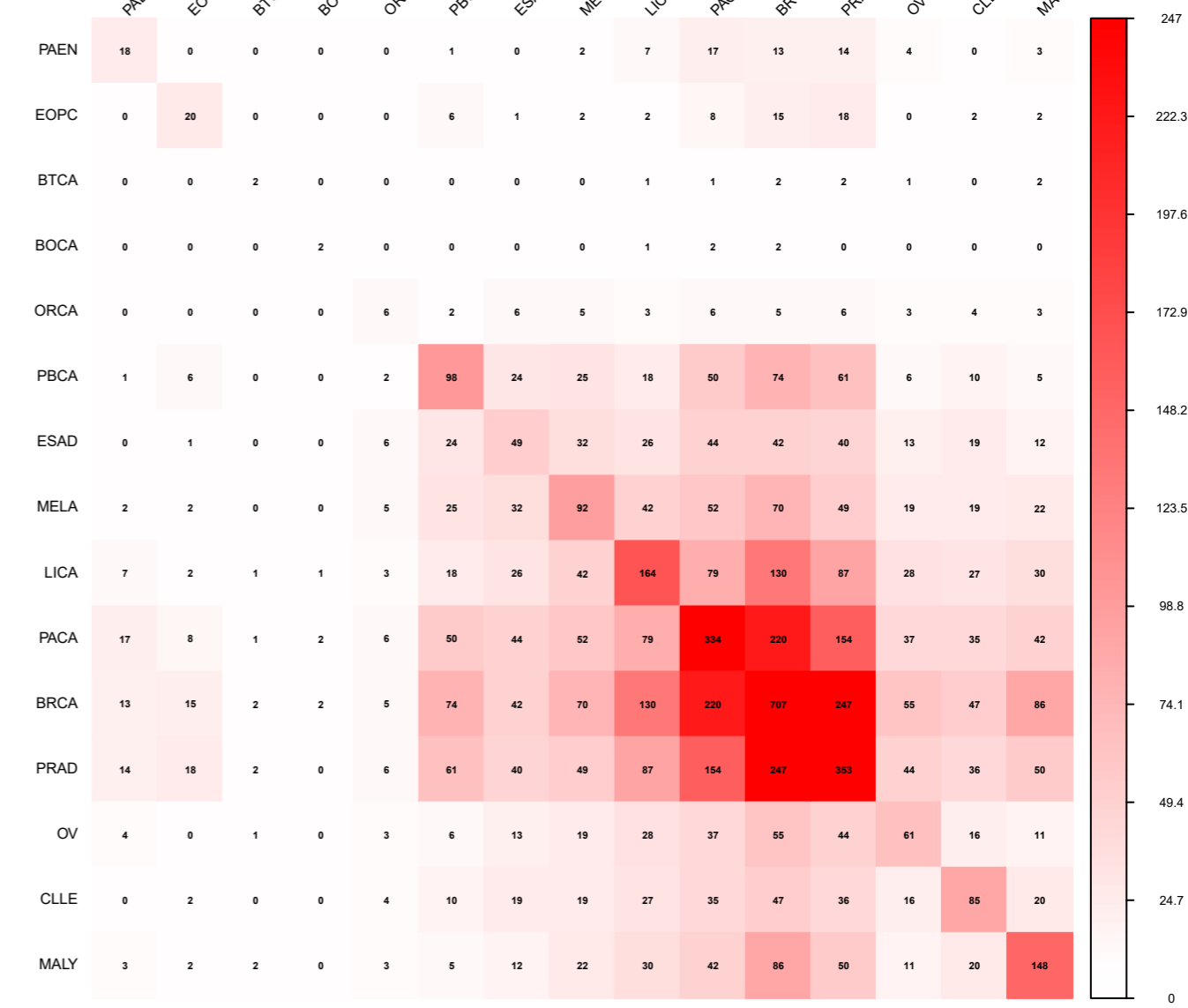

Supplement: gkaa1033_Supplemental_Files [file gkaa1033_supplemental_files.zip › Figure S3.pair-wise.onco.pathway.overlap.hclust.cluster.pdf]

# Supplemental Figure 4

**A**

TCGA cohorts

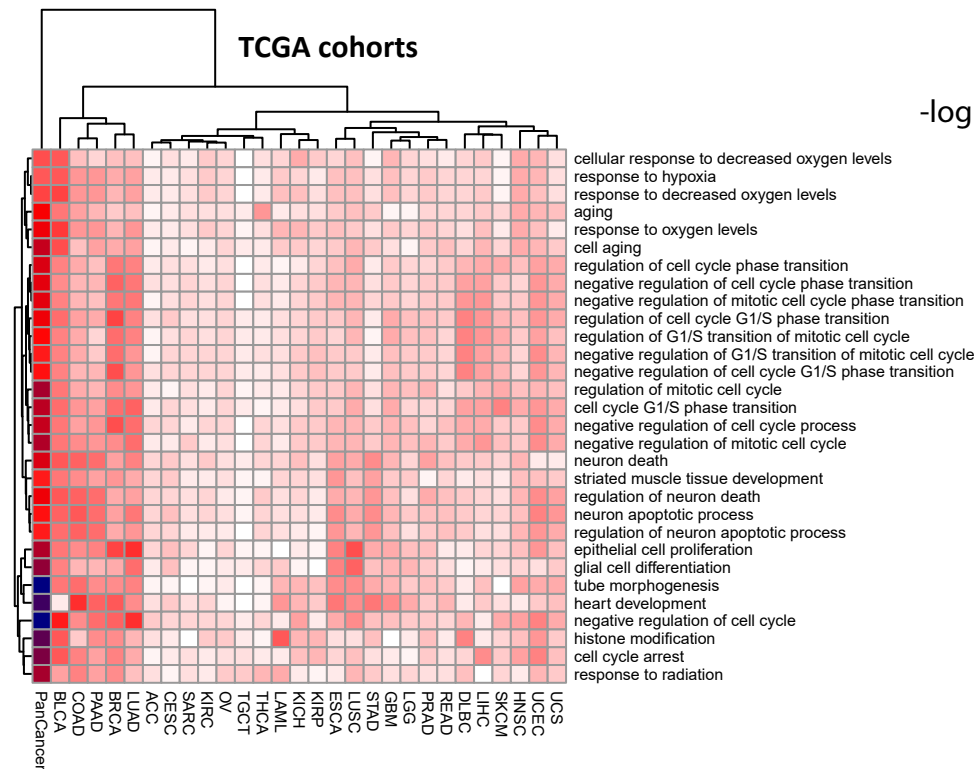

**B**

ICGC cohorts

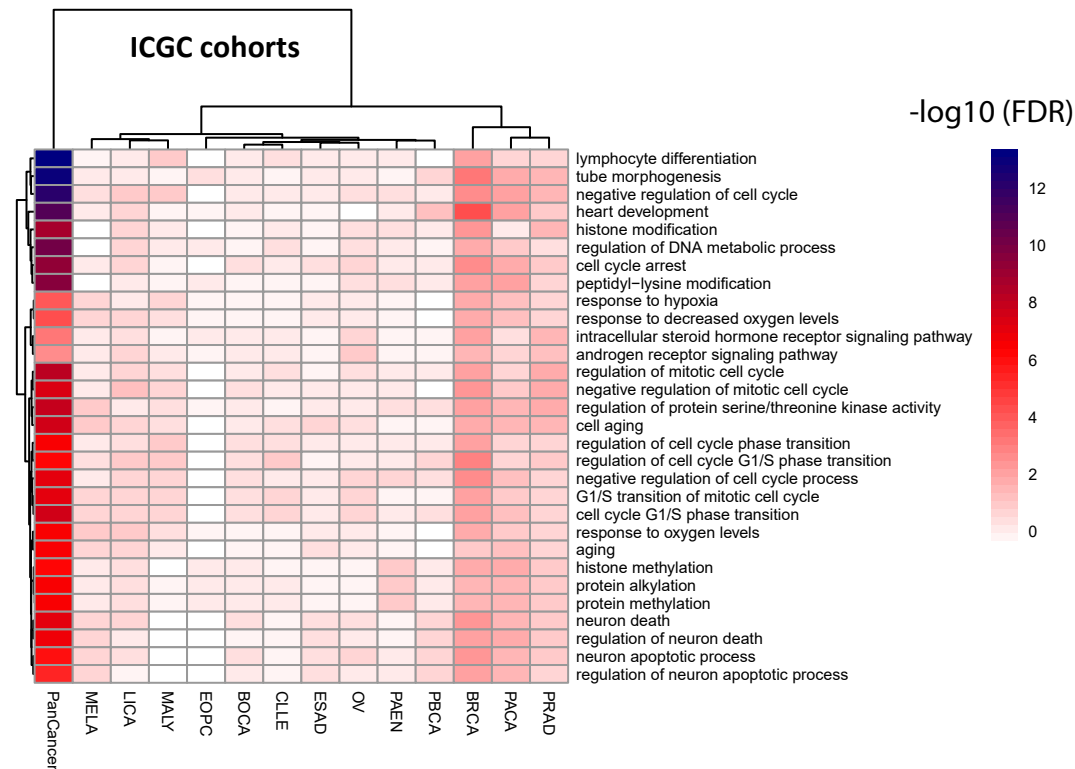

Supplement: gkaa1033_Supplemental_Files [file gkaa1033_supplemental_files.zip › Figure S4. BP.top30.heatmap.clustering_method.complete.pdf]

Supplemental Figure 5

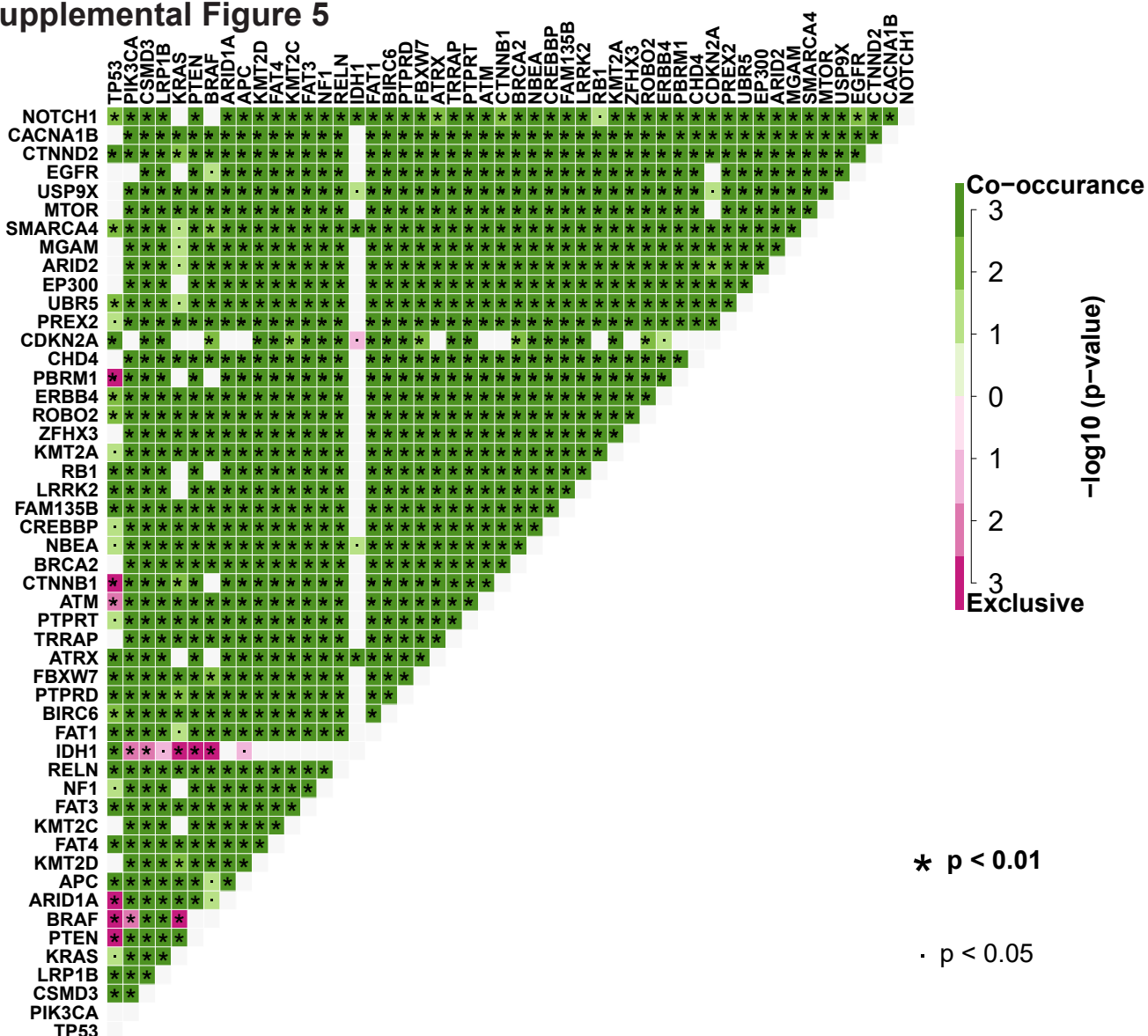

Supplement: gkaa1033_Supplemental_Files [file gkaa1033_supplemental_files.zip › Figure S5. TCGA.PanCancer.Interactions.pdf]

# Supplemental Figure 6

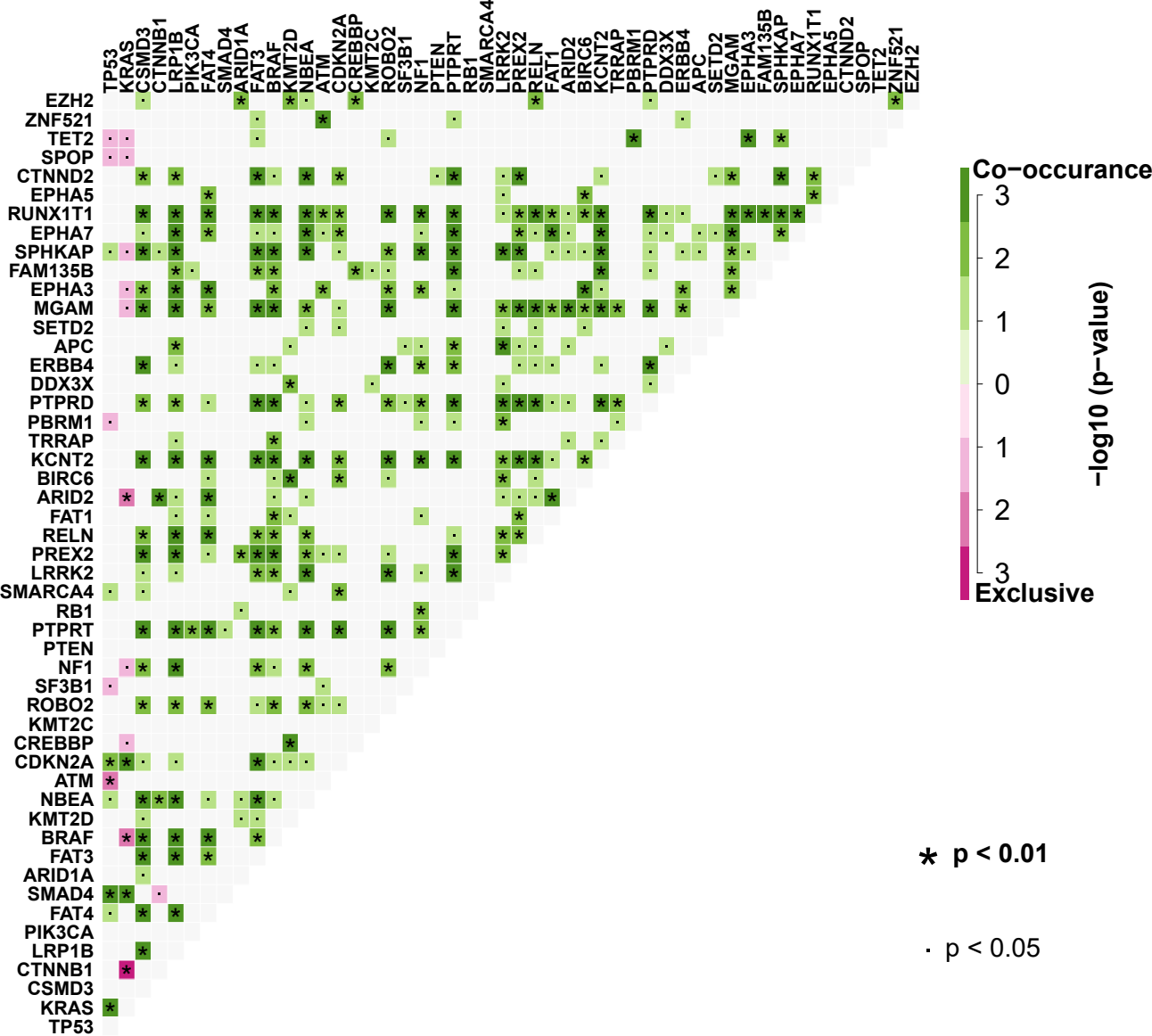

Supplement: gkaa1033_Supplemental_Files [file gkaa1033_supplemental_files.zip › Figure S6. ICGC.PanCancer.gene.Interactions.pdf]

Supplemental Figure 7

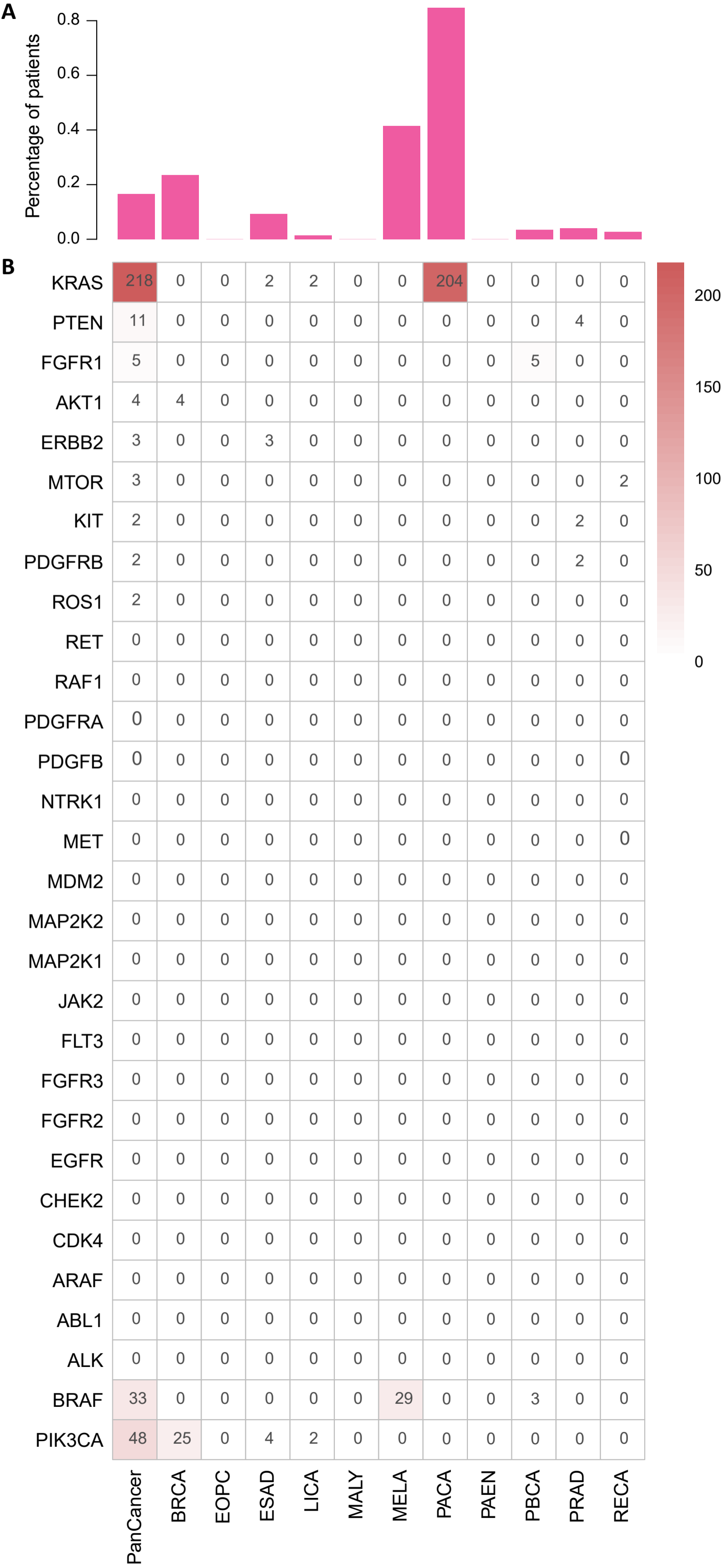

Supplement: gkaa1033_Supplemental_Files [file gkaa1033_supplemental_files.zip › Figure S7. ICGC.driver_mutation.patient_percentage.pdf]
